# How to Adapt Anesthetic Human Resources to Health Emergencies Such as the COVID-19 Outbreak: Replacing a Pre-anesthetic Consultation With a Questionnaire in a University Obstetric Unit

**DOI:** 10.3389/fmed.2022.770199

**Published:** 2022-05-18

**Authors:** Emilie Boussac, Charlene Gery, David Desseauve

**Affiliations:** ^1^Department of Surgical and Anesthesiology Services, Lausanne University Hospital (CHUV), Lausanne, Switzerland; ^2^Department “Woman-Mother-Child”, Lausanne University Hospital (CHUV), Lausanne, Switzerland

**Keywords:** anesthesiology, consultation, obstetrics, preoperative, questionnaire

## Abstract

To manage referrals to the pre-anesthetic consultation during the COVID-19 pandemic, a screening questionnaire was created and sent to parturients with anesthetic risk during the pre-anesthetic consultation. This innovative approach enabled the redistribution of medical anesthetic resources in units that were heavily affected by the pandemic.

## Introduction

Prior to the COVID-19 pandemic, our institution (Lausanne University Hospital) required future mothers to attend a pre-anesthetic consultation (PAC) only in cases of significant medical comorbidities or scheduled surgical intervention. The objectives of PAC are to anticipate the peripartum management of high-risk pregnant patients, select the optimal anesthetic technique, and address the appropriate postoperative follow-up. Prior to the visit, all patients were required, as an initial assessment, to complete a pre-anesthesia questionnaire (PAQ) and determine the indication for a PAC before delivery. This questionnaire is self-administered, but can also be completed with a resource person (e.g., an interpreter in the case of language difficulties).

In some countries, attendance to a PAC is mandatory. Switzerland, however, has no legal equivalence (1, 2). According to the Swiss Society of Anesthesia and Resuscitation (SSAR) standards, attendance to the PAC is optional and at the discretion of the principal care provider. Usually, the indication for an anesthesiology consultation is evaluated by the obstetrician (3). In contrast, for the anesthesia team, it is mandatory to obtain informed consent prior to surgery or intervention (4).

Currently, no PAC pricing exists in the Swiss healthcare insurance system. Thus, healthy patients with uncomplicated pregnancies and planned vaginal deliveries will not attend a PAC.

The COVID-19 pandemic has resulted in many adjustments to our current practices, such as hygiene measures, security of healthcare providers, and identification of high-risk parturients.

The redistribution of competent medical forces to the intensive care units has drastically reduced the availability of the PAC medical staff. To ensure the safety of our patients, the anesthesia and obstetrics teams of the Lausanne University Hospital proposed a screening questionnaire (modified PAQ) that allows for the selection of high-risk parturients and standardization of the referrals to PAC.

## Conception and Implementation into Practice

During obstetric consultations, obstetricians or midwives completed the modified PAQ with patients (Appendix 1 in Supplementary Material). This questionnaire, developed by our anesthesia team, combines the usual pre-anesthetic and bleeding diathesis items (5–10). To facilitate the referral process, some questionnaire items were sub-categorized into “code red” questions. In case of a positive answer to one or more “code RED” items, shared care with the anesthesiologist was implemented with the development of a specific care plan (e.g., complementary investigations, specialist referrals, admission the day before the intervention). On the contrary, if all “code RED” items were answered negatively, the patient was not required to attend a PAC and was assessed on the day of the intervention. A QR code (Appendix 2 in Supplementary Material) was created to facilitate the dissemination of this PAQ to other hospitals.

This PAQ was implemented during the second wave of COVID-19 (March 10, 2021 to June 15, 2021). During this period, 1,040 women gave birth at our institution and all of them were screened. The mean maternal age was 32.7 years, and 45% (*N* = 473) were primiparous. Of the participants, 13% (*N* = 138) gave birth prematurely and thus did not complete the PAQ.

A cesarean section (C-section) was performed in 21% (*N* = 222) of the births. Among them, 40% (*N* = 88) had an elective C-section, and all patients were screened using the PAQ. Following screening, 15% (*N* = 33) of the women were referred for a PAC. Only 5% (*N* = 52) of women who gave birth vaginally required a PAC after screening.

## Conclusion

The current sanitary crisis implies the relocation of resources, with patient safety preservation as the priority. This procedure can be disseminated and assessed in cases with limited sanitary resources.

## Data Availability Statement

The original contributions presented in the study are included in the article/Supplementary Materials, further inquiries can be directed to the corresponding author/s.

## Author Contributions

EB and DD conceived of the presented idea. EB helped CG carry out the questionnaire. All authors discussed the results and contributed to the final manuscript.

## Funding

Open access funding was provided by the University of Lausanne.

## Conflict of Interest

The authors declare that the research was conducted in the absence of any commercial or financial relationships that could be construed as a potential conflict of interest.

## Publisher's Note

All claims expressed in this article are solely those of the authors and do not necessarily represent those of their affiliated organizations, or those of the publisher, the editors and the reviewers. Any product that may be evaluated in this article, or claim that may be made by its manufacturer, is not guaranteed or endorsed by the publisher.

## Supplementary Material

Pre-Anesthetic Questionnaire (PAQ).

QR Code download.
