# Erratum: How to adapt anesthetic human resources to health emergencies such as the COVID-19 outbreak: Replacing a pre-anesthetic consultation with a questionnaire in a university obstetric unit

**DOI:** 10.3389/fmed.2023.1183742

**Published:** 2023-03-21

**Authors:** Frontiers Production Office

**Affiliations:** Frontiers Media SA, Lausanne, Switzerland

**Keywords:** anesthesiology, consultation, obstetrics, preoperative, questionnaire

An omission to the funding section of the original article was made in error. The following sentence has been added: “Open access funding was provided by the University of Lausanne.”

The original article has been updated.

