# Supplementary material for: How to Adapt Anesthetic Human Resources to Health Emergencies Such as the COVID-19 Outbreak: Replacing a Pre-anesthetic Consultation With a Questionnaire in a University Obstetric Unit
Source: Front Med (Lausanne). 2022 May 18;9:770199. doi: 10.3389/fmed.2022.770199 (PMC9158324; doi:10.3389/fmed.2022.770199)
Supplement: Supplementary file 1 [file Table_1.DOCX]

**PRE-ANAESTHESIA QUESTIONNAIRE IN OBSTETRIC**

This questionnaire helps us to identify patients who require a possible anaesthesia consultation.

Collect and scan the document with:

- Letters or specialized exams (cardiologist, lung specialist, blood specialist, …)
- Blood test results :  *complete blood count, platelets,coagulation tests*
- Medical records

PATIENT

INFORMATION

Consultation date: …/…/…

Current weight: ………… kg Height: ………… cm BMI: …………

Expected term: ………… Gravidity: …………. Parity: …………

**OBSTETRICAL HISTORY:** YES NO

Caesarean sections  

Complications (haemorrhage, infections):  

If YES, specify ………………………………………………………………

**SURGICAL/ANAESTHESIC HISTORY:**

Previous surgeries  

If YES, specify what kind of surgery and date:

- ………………………… - ………………………… - …………………………

- ………………………… - ………………………… - …………………………

History of complication of regional anaesthesia

(Multiple attempts, postdural puncture headache, abscess, failure):  

If YES, specify ……………………………………………………………….

History of general anaesthesia:  

Complications (**difficult intubation**, allergies…):  

If YES, specify ……………………………………………………………….

**MEDICAL HISTORY:**

Current treatments:  

If YES, specify:

- ………………………… - ………………………… - …………………………

- ………………………… - ………………………… - …………………………

**Prophylactic / therapeutic anticoagulation**  **** 

**Particular diseases (porphyria, sickle cell anaemia...): ** 

- ………………………… - ………………………… - …………………………

- ………………………… - ………………………… - …………………………

Drug allergies  

If YES, specify the reactions & treatment ………………………………………

Transfusion history  

If YES, specify the context, year, reactions ……………………………………

**Anaemia/ thrombocytopenia:** **** 

Drug consumption: **** 

If YES, daily dose ………………………………………………………

**CARDIOVASCULAR Diseases:**

Arterial hypertension / angina pectoris / heart attack  

Cardiac failure  

Palpitations, arrhythmia  

Personal history of phlebitis / varicose veins  

History of pulmonary embolism   

Others:  

If YES, specify ………………………………………………………………

PULMONARY Diseases:

Asthma  

Sleep apnoea   

If **YES**  Sleep apnoea device  

 Observance   

**Chronic Obstructive Pulmonary Disease, Bronchitis ** 

Other pulmonary diseases  

If YES, specify ………………………………………………………………

DIGESTIVE Diseases:

Hepatic /renal  

Diabetes

**Type I**  

Type II  

Gestational  

Thyroid diseases:  

If YES, specify ………………………………………………………………

NEUROLOGICAL Diseases:

History of epilepsy  

History of **myopathy**  **** 

History of **spinal disease (surgery/scoliosis)**  

Others  

If YES, specify ………………………………………………………………

**BLEEDING DIATHESIS QUESTIONNAIRE:**

- Apart from pregnancy, have you ever consulted a doctor or received any
  treatment for prolonged/unusual bleeding (nosebleed, small cut…)?  
- Do you tend to bruise more than 2cm without shock
  or much more for a minor shock ? **** 
- Have you bled for a long time after tooth extraction
  (requiring consulting with a dentist again) ? **** 
- Have you bled significantly after minor surgery (Adenoids, tonsils,..)? **** 
- Have you consulted a doctor or received a treatment for heavy period
  (Contraceptive pill, iron, tranexamic acid, other)? **** 
- Have you bled abundantly after giving birth? **** 
- Are there any members of your immediate family affected by a coagulation
  disorder (Von Willebrand disease, haemophilia,..) ? **** 

**Call the anaesthesiologist if there is:**

**An answer « YES » in the items marked in red/bold**

**An answer « YES » in the bleeding diathesis questionnaire**
