# Supplementary figures and images for: How to Adapt Anesthetic Human Resources to Health Emergencies Such as the COVID-19 Outbreak: Replacing a Pre-anesthetic Consultation With a Questionnaire in a University Obstetric Unit
Source: Front Med (Lausanne). 2022 May 18;9:770199. doi: 10.3389/fmed.2022.770199 (PMC9158324; doi:10.3389/fmed.2022.770199)

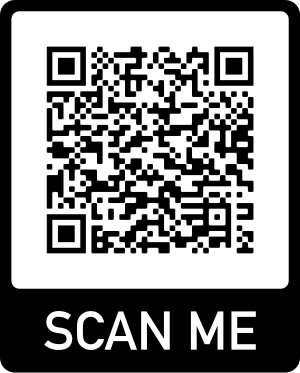

Supplement: Supplementary file 2 [file Image_1.PNG]
